# Alanyl-glutamine protects the intestinal barrier function in trained rats against the impact of acute exhaustive exercise

**DOI:** 10.1590/1414-431X20209211

**Published:** 2020-04-17

**Authors:** A.K.L. Freitas, M.T.B. Silva, C.M.S. Silva, M.M.G. Prata, F.A.P. Rodrigues, R.J.B. Siqueira, A.A.M. Lima, A.A. Santos, A. Havt

**Affiliations:** 1 Departamento de Fisiologia e Farmacologia, Faculdade de Medicina, Universidade Federal do Ceará, Fortaleza, CE, Brasil; 2 Departamento de Educação Física, Universidade Federal do Piauí, Teresina, PI, Brasil; 3 Departamento de Educação Física e Esporte, Instituto Federal de Educação, Ciência e Tecnologia do Ceará, Fortaleza, CE, Brasil

**Keywords:** Claudin-2, Intestinal permeability, Lactulose-mannitol test, Physical exercise, Tight junctions

## Abstract

Strenuous exercise triggers deleterious effects on the intestinal epithelium, but their mechanisms are still uncertain. Here, we investigated whether a prolonged training and an additional exhaustive training protocol alter intestinal permeability and the putative effect of alanyl-glutamine (AG) pretreatment in this condition. Rats were allocated into 5 different groups: 1) sedentary; 2 and 3) trained (50 min per day, 5 days per week for 12 weeks) with or without 6 weeks oral (1.5 g/kg) AG supplementation; 4 and 5) trained and subjected to an additional exhaustive test protocol with or without oral AG supplementation. Venous blood samples were collected to determine gasometrical indices at the end of the 12-week protocol or after exhaustive test. Lactate and glucose levels were determined before, during, and after the exhaustive test. Ileum tissue collected after all experimental procedures was used for gene expression analysis of Zonula occludens 1 (ZO-1), occludin, claudin-2, and oligopeptide transporter 1 (PepT-1). Intestinal permeability was assessed by urinary lactulose/mannitol test collected after the 12-week protocol or the exhaustive test. The exhaustive test decreased pH and base excess and increased pCO_2_. Training sessions delayed exhaustion time and reduced the changes in blood glucose and lactate levels. Trained rats exhibited upregulation of PEPT-1, ZO-1, and occludin mRNA, which were partially protected by AG. Exhaustive exercise induced intestinal paracellular leakage associated with the upregulation of claudin-2, a phenomenon protected by AG treatment. Thus, AG partially prevented intestinal training adaptations but also blocked paracellular leakage during exhaustive exercise involving claudin-2 and occludin gene expression.

## Introduction

Physical activity has been prescribed for patients with gut dysmotility to improve their health (1). However, physical exercises may impair the gastrointestinal tract physiology, eliciting vomit, diarrhea, bloody stool, and gastro-esophageal reflux, especially after prolonged and strenuous activity involving heat stress (2). The impact of physical exercise on human gut motor behavior is still uncertain. There is conflicting evidence concerning its effects on gastric emptying rate (GER) (3). Such discrepancies may be explained by different experimental conditions including exercise protocols, nature of the test meal, and duration of the post-prandial interval for GER analysis. We previously reported that acute high-intensity exercise delayed the GER of a liquid test meal in awake rats, a phenomenon prevented by bicarbonate administration (4). Although the etiology of exercise-induced gastrointestinal tract dysmotility appears to be multifactorial, gut ischemia is considered the major pathophysiological mechanism (5). Strenuous physical exercise leads to blood volume redistribution, shunting blood flow away from the splanchnic area. Therefore, gut ischemia leads to tissue hypoxia, local depletion of adenosine triphosphate (ATP), acidosis, local inflammation, and formation of reactive oxygen species (6,7). Physical stress is associated with a high risk of intestinal mucosal integrity loss and bacterial translocation (8). However, the precise mechanisms of increased gut permeability, in this context, are still unknown (7,9).

Glutamine is a major substrate for enterocytes and cells of the mucosal immune system and accelerates the repair of damaged intestinal mucosa and barrier function (10,11). Glutamine, however, presents limited solubility and has a tendency to hydrolyze into potentially toxic glutamate. Linking glutamine to alanine has solved both drawbacks. We previously observed that alanyl-glutamine (AG) is stable, highly soluble, well tolerated, effective for sodium transport, and a key component for repairing intestinal injury both in laboratory animals and human subjects (12).

The present study evaluated whether periodized training and exhaustive exercise would alter intestinal permeability in a rat model and the putative effects of AG pretreatment in the gut function. We hypothesized that AG would be able to protect intestinal cells against leakage, presumably caused by strenuous exercise.

## Material and Methods

### Animals and experimental design

Male Wistar rats (300–350 g) were supplied by the Central Vivarium of the Federal University of Ceará (Brazil). All experimental protocols were conducted in accordance with the principles for the ethical use of animals for scientific research regulated by CONCEA (National Council for Control of Animal Experimentation), and performed after approval by the Ethical Committee for Animal Use of the Federal University of Ceará, Brazil (protocol 13/2013).

Rats were randomly divided into five groups (n=8 each): sedentary group (S); trained group (50 min per day, 5 days per week for 12 weeks) (T); trained group (50 min per day, 5 days per week for 12 weeks) supplemented orally with AG for the last 6 weeks of swimming sessions (T-AG); trained group (50 min per day, 5 days per week for 12 weeks) subjected to an exhaustive test (ET) that took place after the 12-week swimming protocol; and trained group (50 min per day, 5 days per week for 12 weeks) supplemented orally with AG for the last 6 weeks of swimming session and subjected to an exhaustive test that took place after the 12-week swimming protocol (ET-AG). L-Alanyl-glutamine (Ajinomoto^®^, Brazil) was given by gavage (1.5 g/kg) at the end of each daily swimming session for the last six weeks of the training protocol (13,14) (Supplementary Figure S1). Animals without supplementation received only the vehicle (water) by gavage.

The physical training protocol consisted of swimming exercise in a cylindrical tank (60 cm diameter, 50 cm depth, 30±1°C water temperature). All animals underwent an adaptive period. This adaptive period was done for five consecutive days, in which the animals swam freely for 10, 20, 30, 40, and 50 min, respectively. After the adaptive period, rats from T, T-AG, ET, and ET-AG groups were subjected to the training period, which consisted of 50-min daily swimming sessions, from Monday to Friday for 12 weeks (15). Animals of the sedentary group were placed from Monday to Friday for 12 weeks for 10 min in tanks with enough water to immerse their paws (5 cm).

The rats of ET and ET-AG groups, exactly 24 h after the last session of the 12-week swimming protocol, were subjected to an additional swimming exhaustive test, without extra load (13 [Bibr B14],^16^,^17^). Uncoordinated movements and a contorted body characterized the physical collapse in the exhaustive test, such that the rats could not keep their nostrils above the water for 10 s. The extent of exhaustion time (in min) was determined for ET and ET-AG groups. Blood samples were collected from the animals' tails after performing a tiny cut of its tip, according to the technique described by Rogero et al. (13). The first sample was collected immediately before starting the exhaustive test. The second sample was collected exactly two hours after the beginning of the exhaustive test. The third one was collected right after the end of the exhaustive test. These samples served for glucose and lactate analysis performed by Accutrend Plus instrument (Roche Diagnostics, Germany).

All animals were gavage-fed with a lactulose/mannitol solution (200 and 50 mg/mL, respectively) (18). The S, T, and T-AG groups received lactulose/mannitol solution after the last day of the 12-week protocol. The animals in ET and ET-AG groups received the solution after the exhaustive test. After the administration of the lactulose/mannitol solution, all animals were individually housed in metabolic cages for 24 h for urine collection. Finally, all rats were euthanized under anesthesia (pentobarbital sodium 50 mg/kg) followed by exsanguination. After death was clinically detected, ileum tissue was collected for RNA expression analysis.

### Blood gas analysis

Under anesthesia, we collected a sample of venous blood from the retro-orbital plexus to measure pH, carbon dioxide partial pressure (pCO_2_), oxygen partial pressure (pO_2_), base excess (BE), O_2_ saturation (SatO_2_), and bicarbonate (HCO_3_^-^) using a gasometer (Cobas b121, Roche Diagnostics, Germany). This procedure was executed right after the last swimming session for groups S, T, and T-AG and right after the exhaustive test for ET and ET-AG.

### Intestinal permeability evaluation

Intestinal permeability was evaluated by analyzing urinary lactulose and mannitol levels (18). After returning from anesthesia for blood collection for gasometrical analysis, rats were gavage-fed with a lactulose (200 mg/mL) and mannitol (50 mg/mL) solution (1 mL/kg) and kept individually in metabolic cages for 24 h. During this period of 24 h, urine was collected in a flask containing 25 µL of chlorhexidine solution (40 mg/mL). Total urine volume was recorded, and two aliquots were stored at −20°C (17). Each urine sample (50 µL) was mixed with 50 µL of a melibiose solution (3.6 mM) and diluted into 2.9 mL of double-distilled water. After centrifugation at 562 *g* for 10 minutes at 4°C and filtration, a sample was used for sugar determination by high-performance liquid chromatography (19).

### mRNA expression of intestinal transporters and tight junctions

Total RNA was extracted from the ileum using the RNeasy Lipid Tissue Mini Kit (Qiagen, Germany) according to the manufacturer's instructions and then subjected to cDNA synthesis using the iScript cDNA Synthesis Kit (Bio-Rad, USA). The mRNA expression of the tight junction (Zonula occludens-1, claudin-2, and occludin) and oligopeptide transporter 1 (PepT-1) was assayed by real-time quantitative polymerase chain reaction (qPCR) using the CFX96 Touch Detection System (Bio-Rad). The tyrosine 3-monooxygenase/tryptophan 5E-monooxygenase activation protein zeta polypeptide (YWHAZ) served as housekeeping gene (20). DNA primers for all the analyzed genes (Supplementary Table S1) were designed on the basis of mRNA sequences obtained from the National Centre for Biotechnology Information (http://www.ncbi.nlm.nih.gov). The data were obtained using CFX Manager 3.0 software (Bio-Rad), based on the values of the threshold cycle, in which the observed fluorescence is 10-fold higher than the basal fluorescence for each qPCR assay. The mRNA expression was determined by applying the 2^-ΔΔCT^ mathematical method (21).

### Statistical analysis

Data are reported as means±SD. One-way analysis of variance (ANOVA) followed by Bonferroni's *post hoc* test was used for parametric data. mRNA expression data were evaluated by Mann-Whitney's test. P<0.05 was considered statistically significant.

## Results

### Parameters of exercise intensity

**Figure 1:**
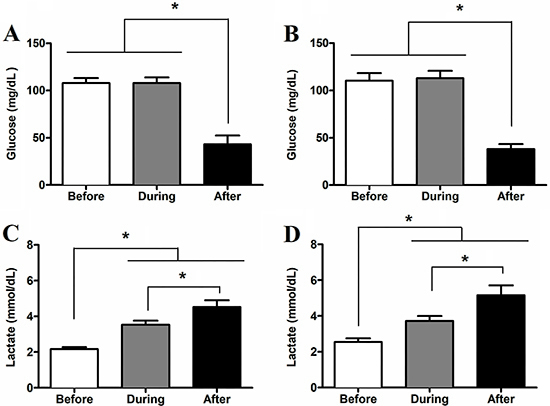
Serum glucose (**A** and **B**) and lactate (**C** and **D**) concentration measured before, during (50 min), and after the exhaustive test. Data for ET groups (trained animals subjected to an exhaustive test without supplementation) are shown in graphs **A** and **C**. Data for ET-AG groups (trained animals subjected to exhaustive test and supplemented with alanyl-glutamine) are shown in graphs **B** and **D**. The data (n=8) are reported as means±SD. *P<0.05, ANOVA followed by Bonferroni *post hoc* test.

Figure 1 shows the serum glucose and lactate levels of rats subjected to the exhaustive test. Serum glucose levels decreased (P<0.05) in blood samples collected immediately after the exhaustive test, regardless of AG supplementation. There was no difference in glucose levels between ET and ET-AG groups regardless of swimming duration during the exhaustive test. Rats of ET and ET-AG groups swam for 248.9±57.6 min and 246.6±52.4 min, respectively. Animals of the ET group showed glucose levels of 107.9±5.49, 107.8±6.08, and 43.2±9.24 mg/dL before, during, and after the exhaustive test, respectively (Figure 1A). Animals from the ET-AG group showed glucose levels of 110.4±7.89, 113.2±7.60, and 38.0±5.33 mg/dL before, during, and after the exhaustive test, respectively (Figure 1B). Compared with their respective basal levels, serum lactate increased (P<0.05) during and after the exhaustive test for both the ET and ET-AG groups. Before, during, and after the exhaustive test, the ET rats showed serum lactate levels of 2.1±0.10, 3.5±0.24, and 4.5±0.39 mmol/L, respectively (Figure 1C). Animals in ET-AG group presented lactate levels of 2.5±0.21 mmol/L before, 3.7±0.28 mmol/L during, and 5.1±0.55 mmol/L after the exhaustive test (Figure 1D). AG supplementation did not affect lactate metabolism (Figure 1D).

### Gasometric parameters

The gasometric data are presented in Table 1. Compared to the S group, animals from the T-AG, ET, and ET-AG groups had lower pH (P<0.05). The animals of the ET-AG group had lower BE compared to the S group (P<0.05). Only the animals subjected to the exhaustive test had significantly higher pCO_2_ compared to the S group. In addition, animals from the ET group also had higher pCO_2_ than animals of the T group (P<0.05). Animals from the ET and ET-AG groups had lower (P<0.05) SatO_2_ compared to sedentary animals.

**Table 1:** Gasometric analysis results.

Variables	Training				
	S	T	T-AG	ET	ET-AG
pH	7.36±0.03	7.33±0.03	7.30±0.04*	7.30±0.02*	7.26±0.01*
BE (mmol/L)	-1.28±1.10	-2.78±0.55	-3.28±1.20	-2.88±2.10	-4.17±1.40*
HCO_3_^-^ (mmol/L)	23.82±1.23	23.72±1.28	23.64±0.96	24.60±1.51	24.98±1.74
pCO_2_ (mmHg)	41.18±3.47	45.02±2.48	45.12±3.07	50.33±2.7*^#^	49.92±2.32*
pO_2_ (mmHg)	58.99±5.37	53.81±2.34	56.10±5.45	55.03±4.00	56.78±5.82
SatO_2_ (%)	75.30±2.91	67.97±2.98	72.03±4.77	65.74±4.84*	64.96±7.27*

S: sedentary; T: trained; T-AG: trained and supplemented with alanyl-glutamine; ET: trained and subjected to an exhaustive test; ET-AG: trained group supplemented with alanyl-glutamine and subjected to an exhaustive test; BE: base excess; HCO_3_^-^: bicarbonate; pCO_2_: carbon dioxide partial pressure; pO_2_: oxygen partial pressure: SatO_2_: oxygen saturation. Data are reported as means±SD. *P<0.05 compared to S group; ^#^P<0.05 compared to T group (ANOVA followed by Bonferroni *post hoc* test).

### Impact of AG supplementation on intestinal permeability

**Figure 2:**
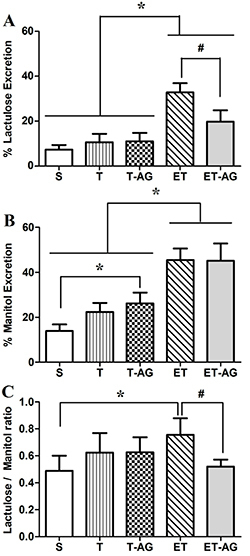
Intestinal permeability was evaluated by the percentage of lactulose, mannitol, and lactulose/mannitol ratio. Animals were subdivided into 5 groups: sedentary without supplementation (S), trained animals without supplementation (T), trained animals supplemented with alanyl-glutamine (T-AG), trained animals subjected to an exhaustive test without supplementation (ET), and trained animals subjected to an exhaustive test and supplemented with alanyl-glutamine (ET-AG). The data are reported as means±SD (n=8). *P<0.05 compared with S; ^#^P<0.05 compared with ET (ANOVA followed by Bonferroni *post hoc* test).

Figure 2A shows that animals of the ET group increased (P<0.05) lactulose excretion compared to the S, T, and T-AG groups (32.83±4.12 *vs* 7.34±2.05, 10.59±3.75, and 11.03±3.76%, respectively). AG treatment attenuated (P<0.05) this phenomenon in the ET-AG group (32.83±4.12 *vs* 19.73±5.14%). We also observed an increase of the mannitol urinary excretion for the T-AG animals compared to sedentary animals (Figure 2B). The ET group showed higher (P<0.05) mannitol levels (45.49±5.12%) than the S, T, and T-AG groups (13.91±2.91, 22.33±4.06, and 26.18±4.78%, respectively). In addition, ET-AG animals presented mannitol excretion levels of 45.07±7.75 showing that AG supplementation did not significantly alter mannitol excretion. Figure 2C shows that the lactulose/mannitol excretion ratio was higher (P<0.05) in the ET group compared to the S group (0.76±0.12 *vs* 0.49±0.11%). It was also higher (P<0.05) in the ET group compared to the ET-AG (0.76±0.12 *vs* 0.52±0.05%).

### Gene transcription of cellular intestinal transporters and tight junction

**Figure 3:**
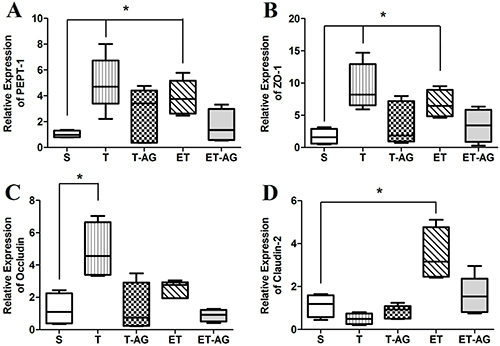
Relative mRNA expression of (**A**) peptide transporter-1 (PEPT-1), (**B**) zonula occludens-1 (ZO-1), (**C**) occludin, and (**D**) claudin-2. Animals were subdivided into 5 groups: sedentary without supplementation (S), trained animals without supplementation (T), trained animals supplemented with alanyl-glutamine (T-AG), trained animals subjected to an exhaustive test without supplementation (ET), and trained animals subjected to an exhaustive test and supplemented with alanyl-glutamine (ET-AG). The data are reported as median, minimum and maximum, and interquartile range (n=8). *P<0.05 compared with the S group (Mann-Whitney test).

**Figure 4:**
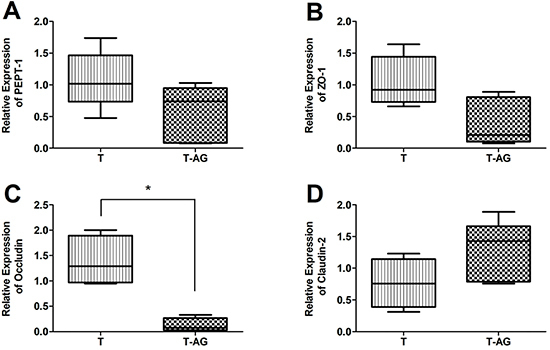
Relative mRNA expression of (**A**) peptide transporter-1 (PEPT-1), (**B**) zonula occludens-1 (ZO-1), (**C**) occludin, and (**D**) claudin-2 between trained animals without supplementation (T group) and trained animals supplemented with alanyl-glutamine (T-AG). The data are reported as median, minimum and maximum, and interquartile range (n=8). *P<0.05 compared with the T group (Mann-Whitney test).

**Figure 5:**
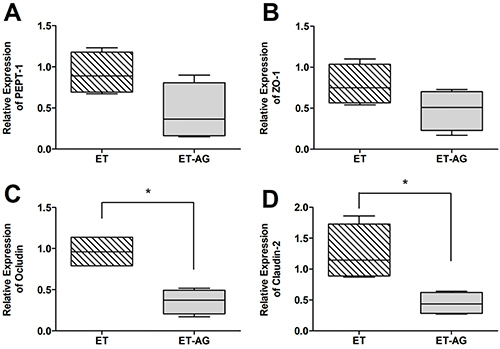
Relative mRNA expression of (**A**) peptide transporter-1 (PEPT-1), (**B**) zonula occludens-1 (ZO-1), (**C**) occludin, and (**D**) claudin-2 between trained animals submitted to an exhaustive test without supplementation (ET group) and trained animals submitted to an exhaustive test supplemented with alanyl-glutamine (ET-AG). The data are reported as median, minimum and maximum, and interquartile range (n=8). *P<0.05 compared with the ET group (Mann-Whitney test).

Analyzing oligopeptide transporter 1 (PepT-1) transcription, T and ET groups exhibited a significant upregulation (1.03±0.27 *vs* 4.99±2.1, P=0.016 and 3.87±1.35, P=0.016, respectively) compared to S animals (Figure 3A). In addition, T-AG and ET-AG showed no statistical differences compared to the sedentary animals (2.59±2.08 and 1.64±1.29, respectively). The same behavior is seen in Figures 4A and 5A, in which there were no differences comparing T *vs* T-AG (1.08±0.45 and 0.56±0.45, respectively) or ET vs ET-AG (0.92±0.25 and 0.44±0.35, respectively).

For gene expression analysis of the tight junction zona ocludens-1 (ZO-1), Figure 3B shows upregulation in the animals from the T and ET groups when these groups were compared to the S group (9.44±3.52, P=0.016 and 6.76±2.15, P=0.029 *vs* 1.71±1.21), respectively. When we compared T-AG and ET-AG (Figure 3B) to the S group, there were no significant differences (3.63±3.33 and 3.38±2.55 *vs* 1.71±1.21, respectively). To determine if AG was completely reverting ZO-1 expression, we compared the group T *vs* T-AG (Figure 4B) and ET *vs* ET-AG (Figure 5B). However, we noticed that there was no statistical difference among these two comparisons (1.05±0.39 *vs* 0.41±0.37 and 0.79 ±0.25 *vs* 0.48±0.25, respectively).

After analyzing the ileum gene expression of occludin (Figure 3C), we observed that when group S was compared to all others (T, T-AG, ET, and ET-AG) only animals from group T were significantly upregulated (1.25±0.99 *vs* 4.87±1.74, P=0.029; 1.30±1.53; 2.51±0.52; 0.88±0.37, respectively). These data showed that AG reverted the expression of occludin in the T-AG group. To verify if this reversion was complete, we compared group T with T-AG (Figure 4C). Our data showed that T animals were also statistically different from T-AG animals, indicating that there was a complete reversion of occludin expression after AG supplementation (1.38±0.49 *vs* 0.13±0.14, P=0.03). In addition, occludin expression of group ET was statistically different from group ET-AG (0.96±0.20 *vs* 0.36±0.15, P=0.03; Figure 5C).

As shown in Figure 3D, when the sedentary group was compared to all other groups (T, T-AG, ET, and ET-AG), claudin-2 transcription was upregulated only for the ET group (1.12±0.53 *vs* 0.50±0.25, 0.83±0.31, 3.46±1, P=0.03, and 1.58±0.88, respectively). To verify if AG supplementation was really reverting the mRNA expression of claudin-2 (Figure 5D), we performed the comparison of ET *vs* ET-AG. A statistical difference was found between ET and ET-AG (1.26±0.45 *vs* 0.44±0.18, P=0.03). However, we could not see any statistical difference between groups T and T-AG for claudin-2 mRNA expression (0.77±0.39 *vs* 1.27±0.47).

## Discussion

The present study showed that 12 weeks of periodized training substantially enhanced the performance of rats subjected to an exhaustive test protocol. After a period of physical training, the rats subjected to long-term exhaustive exercise showed increased paracellular intestinal permeability, which was attenuated by AG pretreatment. In addition, AG mediated other effects in animals that were not submitted to the exhaustive test.

The impact of physical exercise on the gastrointestinal tract is well known. In this sense, we recently reported that acute exercise delays the gastric emptying rate in rats, a phenomenon prevented by bicarbonate treatment. In addition, extracellular acidosis *in vitro* inhibits cholinergic neurotransmission in the rat gastric fundus by selectively influencing the Gq/11 protein-signaling pathway (22,23). Now, we report for the first time the morphofunctional modifications in the intestinal barrier, such as increased intestinal permeability associated with changes in the transcription of a tight junction (claudin-2) in a physically exhaustive test, which was prevented by AG treatment.

The impact of exercise on gut permeability was assessed by the non-invasive lactulose/mannitol assay, which is considered a gold standard method (24). The intestinal transport of mannitol and lactulose are directly related to transcellular and paracellular permeability, respectively. Thus, damage to epithelial cells and tight junctions are associated with decreased mannitol and increased lactulose absorption (25). In the present work, both lactulose and mannitol excretion increased in trained rats subjected to exhaustive exercise. In pre-clinical and clinical studies, high urinary excretion of both probes is indicative of the leaky gut syndrome (26,27). It is suggested that during maximum exercise, the blood is shunted away from the viscera, and splanchnic blood flow can decrease by as much as 80% (28). Therefore, strenuous exercise can impair the gastrointestinal blood flow, making the gut mucosa susceptible to ischemic injury and increasing the intestinal permeability. Physical exercise at a 70% VO_2_-max intensity was also recently shown to increase the intestinal permeability in humans (29). In the present study, the trained animals subjected to the exhaustive test swam for almost 4 h, and the stress and lack of splanchnic nutrition could have contributed to increase intestinal permeability. Our finding reinforces a recent study, where runners demonstrated increase intestinal permeability, regardless of the presence of gastrointestinal symptoms (30). In addition, both groups subjected to exhaustive exercise showed signs of physical stress related to acidosis and hypercapnia. Thus, we suggest that the duration of this stress for a longer period of time in trained animals could explain their increase in gut permeability.

The tight junction proteins have important functions for intestinal epithelial tissue, and several pathophysiological conditions modify its morphofunctional configuration (31). In the present study, ZO-1 and occludin mRNA up-regulation could be related to morphological adaptations promoted by physical exercise, but not associated with increasing gut permeability. In the present study, training induced occludin and ZO-1 mRNA up-regulation. This may explain why these animals did not exhibit alterations in gut permeability. The overexpression of these mRNAs may indicate higher resistance of the tight junction assembly (29). ZO-1 serves as an intermediary in the contact between occludin and the actin cytoskeleton, and the upregulation of ZO-1 and occludin mRNA would confirm the hypothesis of higher tight junction resistance. These morphological adaptations promoted by physical exercise were somehow regulated by AG supplementation. Further studies are necessary to clarify the exact molecular mechanism in which AG interfered on tight junction mRNA expression.

However, training did not interfere with claudin-2 expression. Claudin-2 knockout mice showed small intestine paracellular damage with malabsorption of nutrients and electrolytes (32). Claudin proteins are considered primary seal-forming proteins, and have the ability to polymerize into linear fibrils, (33). Loss of claudin-2 expression has been shown to compromise paracellular intestinal absorption of essential nutrients resulting in premature death of mice (32). We suggest that claudin-2 mRNA upregulation possibly contributed to the increased intestinal permeability observed in the present study. In addition, increased protein expression of claudin-2 is associated with lower transepithelial electrical resistance and higher cation selectivity (34). Recently, intestinal leakage has been correlated with increased gene transcription of claudin-2 and critical modifications of the intestinal microbiota (35). Moreover, the increased intestinal permeability in runners is strongly linked to microbiome modifications during prolonged exercise (36).

One goal in the present study was to determine whether chronic AG treatment would be beneficial to intestinal permeability after a swim challenge. Glutamine is the main fuel used by intestinal mucosa cells and other rapidly proliferating tissues (37). Because of its superior physicochemical solubility and favorable thermal stability in acid medium (38), glutamine was replaced by dipeptide AG. It is well established that glutamine absorption is enhanced after the ingestion of AG compared to the free amino acids (39). This dipeptide is transported into enterocytes by PepT-1. We found that PepT-1 mRNA was upregulated in the groups that underwent training (T and ET groups). It is conceivable that physical activity requires adaptations for more nutrients to be absorbed, and an increase in PepT-1 mRNA expression would be one of such adaptations. However, our data suggested that AG supplementation returned the PepT-1 mRNA expression to physiological condition, since the groups that received AG were not different from the sedentary group. Our analysis revealed no differences in PepT-1 mRNA expression between the T and T-AG groups or between the ET and ET-AG groups. Such phenomenon should be investigated further, but intestinal tissue appeared to be more responsive to PepT-1 mRNA expression during exercise since this tissue may need more energetic subtracts provided by AG.

AG treatment did not change the rats' swimming performance; none of the biochemical or gasometrical indices differed in AG-supplemented groups. However, biomarkers of paracellular route were decreased by AG treatment. AG also protected claudin-2 mRNA overexpression. These findings reinforced that AG had modulating mechanisms on the structural proteins present in tight junctions. Some studies showed that glutamine deprivation decreases transepithelial resistance of tight junction proteins, including claudin-1 and occludin (40). Although Noth and coworkers (11) reported that glutamine improved intestinal permeability dysfunction by increasing occludin expression, we did not observe occludin mRNA upregulation in the groups that were subjected to the exhaustive test (11).

In conclusion, chronic AG administration protected intestinal barrier function against the impact of acute exhaustive exercise by reducing intestinal permeability and modulated the mRNA expression of claudin-2. These findings emphasize the importance of AG during physical exercise for gut physiology and support its possible use among endurance athletes.

## Supplementary Material

Click here to view [pdf].
